# Empathic accuracy and oxytocin after tryptophan depletion in adults at risk for depression

**DOI:** 10.1007/s00213-015-4093-9

**Published:** 2015-10-13

**Authors:** Koen Hogenelst, Robert A. Schoevers, Ido P. Kema, Fred C. G. J. Sweep, Marije aan het Rot

**Affiliations:** Department of Psychology, University of Groningen, Grote Kruisstraat 2/1, 9712 TS Groningen, The Netherlands; School of Behavioral and Cognitive Neurosciences, University of Groningen, PO Box 72, 9700 AB Groningen, The Netherlands; University Medical Center Groningen, Department of Psychiatry, University of Groningen, PO box 30.001, 9700 RB Groningen, The Netherlands; University Medical Center Groningen, Department of Laboratory Medicine, University of Groningen, PO box 30.001, 9700 RB Groningen, The Netherlands; Department of Laboratory Medicine, Radboud University Medical Center, P.O. box 9101, 6500 HB Nijmegen, The Netherlands

**Keywords:** Serotonin, Empathy, Cognition, Ecological validity, Major depressive disorder, Oxytocin

## Abstract

**Rationale:**

Major depressive disorder (MDD) has been associated with disturbances in social functioning and in the brain serotonin system. Reduced levels of serotonin may negatively influence social functioning, for example by impairing the recognition of facial emotion expressions.

**Objectives:**

The present study investigated the effect of acute tryptophan depletion (ATD), which reduces brain serotonin, on a related component of social functioning, empathic accuracy (EA), and oxytocin levels.

**Methods:**

Individuals with (FH+) and without (FH−) a family history of MDD participated in a randomized, double-blind, crossover study. On two separate test days, participants ingested tryptophan-deficient and nutritionally balanced amino acid mixtures. Six hours later, they performed an EA task, which involved watching videos of people recounting autobiographical emotional events. While watching, participants continuously rated how these people felt during the recounting. Mood state was repeatedly assessed using the Positive Affect and Negative Affect Schedule and a series of visual analogue scales. Blood samples obtained at baseline and 5 h after mixture ingestion were assessed for tryptophan and oxytocin levels.

**Results:**

ATD decreased circulating levels of tryptophan and oxytocin. Nevertheless, there were no significant effects of ATD on EA or mood in either FH group.

**Conclusions:**

While previous studies have shown that acute reductions in brain serotonin alter the recognition of facial emotion expressions in never-depressed individuals, the present study suggests that empathic abilities may remain unaffected.

**Electronic supplementary material:**

The online version of this article (doi:10.1007/s00213-015-4093-9) contains supplementary material, which is available to authorized users.

## Introduction

Individuals with major depressive disorder (MDD) often experience interpersonal problems (Segrin and Flora 2000). These problems may be due to excessive reassurance seeking and demanding support from others, as well as to reduced levels of prosocial behaviors (Hames, Hagan, and Joiner 2013). Further, depressed individuals often misinterpret facial emotion expressions (Bourke, Douglas, and Porter 2010; Demenescu, Kortekaas, den Boer, and Aleman 2010). Furthermore, their empathic abilities are often impaired (Schreiter, Pijnenborg, and aan het Rot 2013). As impairments in empathy may adversely affect social interactions (Verhofstadt, Buysse, Ickes, Davis, and Devoldre 2008), they represent one potential reason for the interpersonal problems of individuals with MDD (Gadassi, Mor, and Rafaeli 2011).

MDD has also been associated with disturbances in the brain serotonin system (Mann 2013). Experimental lowering of serotonin by acute tryptophan depletion (ATD) induces transient depressive symptoms in recovered MDD patients (for a review, see Young 2013). Moreover, while never-depressed individuals without a family history of MDD (FH−) are generally unaffected by the mood effects of ATD, individuals with a family history of MDD (FH+) often show a mild mood worsening (Booij, Van der Does, and Riedel 2003). These and other studies suggest that serotonin plays a role regulating mood.

Previous ATD studies have also assessed the effects of low serotonin on aspects of social functioning. In healthy volunteers, ATD has been found to decrease cooperative behavior during the prisoner’s dilemma game (Wood, Rilling, Sanfey, Bhagwagar, and Rogers 2006) and increase antagonistic responses in the ultimatum game (Crockett, Clark, Tabibnia, Lieberman, and Robbins 2008), suggesting that reductions in serotonin may impair social functioning. Similar studies have not been performed in recovered MDD patients or in FH+ versus FH− individuals.

Most other studies on the effects of ATD on aspects of social functioning have examined facial emotion recognition (FER). In one study, ATD impaired the recognition of happiness in recovered MDD patients yet enhanced it in controls (Hayward, Goodwin, Cowen, and Harmer 2005). However, in healthy never-depressed individuals, ATD has also been found to impair the recognition of fear (Harmer, Rogers, Tunbridge, Cowen, and Goodwin 2003; Marsh et al. 2006). Indeed, as the “serotonin releaser” 3,4-methylenedioxymethamphetamine (MDMA, “ecstasy”) has also been found to impair fear recognition (Bedi, Hyman, and de Wit 2010; Hysek et al. 2014), the role of serotonin in fear recognition, and more broadly in FER, remains unclear.

Importantly, the ecological validity of most FER tasks is considered low, due to the use of static face stimuli (Hogenelst, Schoevers, and aan het Rot 2015). This precludes an understanding of the role of serotonin in real-life social interactions, where facial expressions are dynamic social signals and therefore important for communicating emotions and facilitating mutual understanding (Ambadar, Schooler, and Cohn 2005; Zaki and Ochsner 2012). Also, to know how a person is feeling, auditory information is at least as important as visual information (Zaki et al. 2009a). Thus, data obtained from laboratory tasks involving dynamic facial expressions and speech may enable better predictions about the role of serotonin in real-world social functioning than data obtained from traditional FER tasks.

Nevertheless, abnormal FER is thought to be at the basis of impaired empathy (Schreiter et al. 2013). If depressed persons perform poorly at recognizing facial emotions, and interpret neutral expressions in a negative way (Bourke et al. 2010; Demenescu et al. 2010), then this may lead them to misread others’ feelings and misunderstand others’ viewpoints (Penton-Voak, Allen, Morrison, Gralewski, and Campbell 2007). In other words, depression-associated FER impairments may contribute to empathic stress, a more affective form of empathy, and to limited empathic accuracy (EA), a more cognitive form of empathy. EA is considered a form of cognitive empathy because it constitutes the ability to accurately infer others’ feelings and thoughts from verbal and nonverbal social information (Ickes 1993). As such, EA is related to perspective taking and theory of mind (Shamay-Tsoory, Aharon-Peretz, and Perry 2009). Brain areas whose activity has been correlated with EA (Zaki et al. 2009b) are known to be affected by ATD (Nishizawa et al. 1997; Williams, Perrett, Waiter, and Pechey 2007), suggesting that EA may also be affected by ATD. EA may be assessed by asking study participants (“perceivers”) to watch video clips of people (“targets”) discussing autobiographical emotional events and rate how the targets were feeling when discussing the events. Correlations between perceivers’ ratings of targets’ feelings and targets’ ratings of their own feelings are then used as a measure of EA. Using naturalistic stimuli increased the ecological validity of this task compared to most FER tasks (Zaki and Ochsner 2009). The task has previously been found to be sensitive to between-person differences in empathic abilities (aan het Rot and Hogenelst 2014; Lee, Zaki, Harvey, Ochsner, and Green 2011; Ripoll et al. 2013). Moreover, the task is sensitive to pharmacological interventions within persons (Bartz et al. 2010). Specifically, intranasal administration of oxytocin improved EA in healthy individuals with higher levels of autism spectrum traits (Bartz et al. 2010).

The present study examined the effects of ATD on EA, oxytocin, and mood in never-depressed individuals with (FH+) and without (FH−) a first-degree family history of MDD. ATD in FH+ individuals can be used to model the effects of low serotonin levels in MDD patients (Booij et al. 2003). FH+ individuals are at increased risk of developing MDD (Sullivan, Neale, and Kendler 2000), possibly because they may exhibit subtle impairments in the processing of emotional stimuli (Mannie, Bristow, Harmer, and Cowen 2007). These impairments may be exacerbated by low brain serotonin, e.g., following ATD (Feder et al. 2011). Further, after ATD FH+ individuals have previously been found to respond differently to negative facial emotion expressions than FH− individuals (van der Veen, Evers, Deutz, and Schmitt 2007). Together, these studies indicate that ATD might also lead FH+ and FH− individuals to be differentially impaired on the EA task. In line with previous studies on the effects of ATD on the recognition of negative facial emotion expressions (e.g., Harmer et al. 2003; aan het Rot, Coupland, Boivin, Benkelfat, & Young, 2010), we expected ATD to reduce EA during videos with negative emotional content. The effects of ATD on mood were examined so we could study the effects of ATD on EA independently of any effects of ATD on mood, which were also expected to be more pronounced in FH+ individuals than in FH− individuals (Booij et al. 2003).

We also expected ATD to reduce circulating oxytocin levels. Serotonin can stimulate oxytocin release through serotonin-1A receptors in the hypothalamus (e.g., Jorgensen et al. 2003; Thompson et al. 2007). Conversely, reductions in serotonin may lower oxytocin release. EA may be affected by low levels of serotonin, low levels of oxytocin, or both.

## Methods

### Participants

The Medical Ethics Committee of the University Medical Center Groningen approved the study. Participants provided written informed consent following an extensive study explanation and were reimbursed 160 euros for their time. The ClinicalTrials.gov identifier is NCT02051530.

Participants were men and women with (FH+) or without (FH−) a first-degree family history of MDD. Similar to previous studies of the effects of ATD on mood and socio-affective processing in FH+ and FH− individuals (Firk and Markus 2008; Klaassen et al. 1999; Neumeister et al. 2002; van der Veen et al. 2007), we recruited 20 individuals in each FH group. FH+ individuals were recruited via patients with a lifetime diagnosis of MDD as verified with their healthcare provider. Inclusion criteria for FH+ individuals were having at least one parent, sibling, or child with MDD, age 18–65 years, no current or past DSM-IV mood disorder including MDD, no other current DSM-IV Axis-I disorder, no current major medical illness, and no use of psychotropic medications. Screening was performed using the Structured Clinical Interview for DSM-IV Axis-I disorders (First et al. 2002) and the family history method by Andreasen et al. (1977). FH− individuals were recruited using advertisements. They were required to have no first- and second-degree relatives with a possible (history of) mood disorder (including suicide). Otherwise, the inclusion criteria were identical. The two groups were matched for gender, age, and education.

Details of the screening phase can be found in the Supplementary Material. Briefly, 44 individuals started the study. Four individuals dropped out (three vomited within the first hour of mixture ingestion on the first test day and one felt sick after completing this day). The results are described for 10 men and 10 women in the FH+ group (all children or siblings of MDD patients, including two sister pairs and one brother pair) and 10 men and 10 women in the FH− group (all from different families). There were no significant group differences (Table 1).

**Table 1:** Participant characteristics

	Group	
	FH− (*n* = 20)	FH+ (*n* = 20)
Age in years, mean (SD)	21.9 (2.2)	22.2 (3.3)
Occupation (% student)	90	95
Body mass index (kg/m^2^), mean (SD)	23.0 (2.1)	22.2 (2.8)
Smoking status (% yes)	30	25

There were no significant group differences by *T* test (*p* > 0.25 for all)

### Study design and overview

Participants received different amino acid mixtures on two separate test days in a double-blind cross-over design, with order of treatment randomized by gender and group in blocks of four. Participants received a nutritionally balanced mixture (B) including tryptophan (2.3 g for men, 1.9 g for women) on 1 day and a tryptophan-deficient but otherwise identical mixture (T−) on the other day. The T− mixtures weighed 103 g for men (Young, Smith, Pihl, and Ervin 1985) and 86 g for women (Ellenbogen, Young, Dean, Palmour, and Benkelfat 1996). Water, chocolate syrup or orange juice, and sodium cyclamate were added just before mixture ingestion.

### Biochemical analysis

Blood samples were collected 15 min before and 5 h after mixture ingestion, using 10-mL Vacutainer tubes containing K_2_EDTA solution as anticoagulant, and within 30 min, centrifuged for 10 min at 2500 g and 4 °C. Plasma was transferred to glass tubes and stored at −20 °C until analysis. Total plasma tryptophan levels were determined using automated online solid-phase extraction-liquid chromatographic-tandem mass spectrometry with deuterated internal standards (de Jong, Smit, Bakker, de Vries, and Kema 2009). Within- and between-assay coefficients of variation (CVs) were 1.7–3.6 % and 1.7–7.0 %, respectively. Reference intervals were 45.5–83.1 pM.

Blood oxytocin analysis was performed using radioimmunoassay (Dumont et al. 2009). The average recovery was 78 ± 6 %. Within- and between-assay CVs were 6.4 and 9.7 %, respectively, at 6.3 pM. The analytical range was 1–90 pM with a sensitivity of 1.5 pM.

### Test measures

Depressive symptoms at baseline were assessed with the Quick Inventory of Depressive Symptomatology (QIDS-SR), obtained from www.ids-qids.org (Rush et al. 2003).

Mood state was measured using the Positive Affect and Negative Affect Schedule (PANAS, Peeters, Ponds, & Vermeeren, 1996) and a list of visual analogue scales (VAS; Bond, James, and Lader 1974). The PANAS included 10 positive affect (PA) items and 10 negative affect (NA) items, all rated on a 5-point Likert scale. The average of the PA and NA item scores were used to calculate PA and NA, respectively. VAS items were rated using 10-cm-long lines ranging from *not at all* to *extreme*. The average of 7 positive mood items and 5 negative mood item scores was used to calculate VAS(+) and VAS(−), respectively.

For the psychometric properties of the PANAS and VAS, see Supplementary Material.

### Empathic accuracy task

EA was assessed using a previously developed computer task, performance on which was found to be negatively associated with autism spectrum traits (aan het Rot and Hogenelst 2014). During the task development phase, described in detail by aan het Rot and Hogenelst (2014), video clips were collected from five male and four female targets. After completing the Berkeley Expressivity Questionnaire (BEQ; Gross and John 1997), targets discussed four negative and four positive autobiographical events while being videotaped. After each event, they rated the overall valence and arousal of their emotions during the recording and subsequently viewed their clips and provided continuous ratings of how they felt while speaking. This was done by moving an on-screen indicator along a 9-point Likert scale, using a rating dial.

Of the 72 stimulus videos, those rated low on arousal or with limited temporal variability in the continuous ratings were discarded. The remaining clips constituted two sets of 20 that were identical in the number of negative and positive clips and in target representation, and comparable in terms of clip length, valance, and arousal. The order of these two sets was randomized over the two test days. On each day, participants watched one of the sets and continuously rated how they thought the targets felt while speaking, using the same rating dial. The order of the clips within each set was randomized per participant, while never more than two positive or negative clips in a row and never the same target more than twice in a row. Participants’ and targets’ raw data were averaged across 5-s periods. Autocorrelations were removed using the Yule-Walker method. EA scores were calculated per participant per video clip using Fisher z-transformed correlations between participant ratings and target ratings.

### Procedures

Test days were separated by at least 3 days (mean 7.8 days). Women were tested in the follicular phase. Testing took place in a private room with standard room lighting turned on and blinds and curtains closed. Before each test day, participants maintained a 24-h low-protein diet. They arrived at the lab at 08:30 am, following an overnight fast. Urine samples were collected to test for recent drug use (Triage™ Panel for Drugs of Abuse, Biosite Incorporated®, San Diego, CA) and, in women, for pregnancy (QuickVue hCG urine test, Quidel, San Diego, CA).

**Fig. 1:**
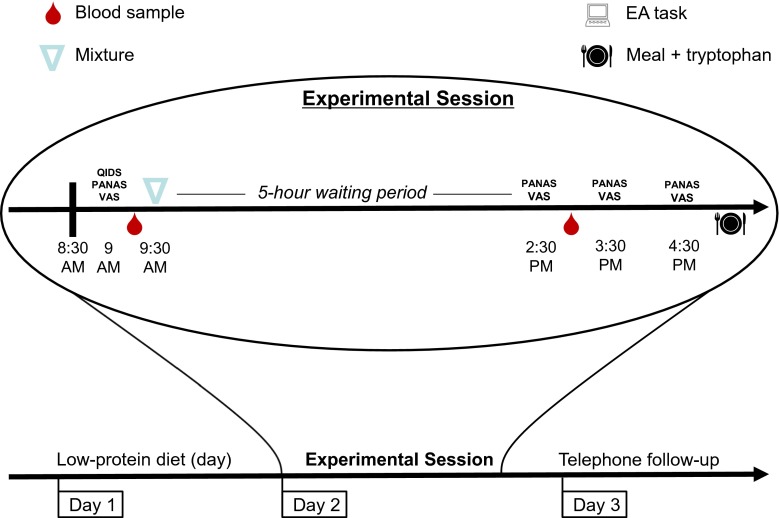
Timeline of events on the two test days for a typical participant

Participants then completed the QIDS-SR, PANAS, and VAS (t0). Approximately 15 min later, a first blood sample was drawn. Subsequently, participants were given up to 30 min to ingest the amino acid mixture. Afterwards, participants were allowed to study, read, and watch a limited set of movies, but not sleep. Besides the mixture and unlimited tap water, no food or beverages were allowed. Mood state was assessed 5, 6, and 7 h after mixture ingestion (t5–t7). Between t5 and t6 (i.e., around 2.30 pm), a second blood draw took place, followed by an assessment of behavioral mimicry and speech (data not presented here). Between t6 and t7, the EA task was administered. After t7, participants were given a sandwich and two capsules with 500 mg tryptophan (Elvitaal^TM^, Lunteren, The Netherlands). After going home, they were followed up by telephone in the evening and the next day. See Fig. 1 for a schematic overview.

### Data analysis

Differences in participant characteristics between the FH groups were examined using *T* tests. Morning baseline data were investigated using general linear modeling (GLM) with group (FH+, FH−) as between-subjects factor and mixture (T−, B) as within-subjects factor. All models included mixture, group, and their interactions. *T* tests and GLM were performed in SPSS 17.0.

The effects of ATD were analyzed using hierarchical linear modeling (HLM) with maximum likelihood estimation to compute the denominator degrees of freedom for the fixed effects tests. HLM was performed with R v3.0.2 (www.r-project.org).

For the primary outcome variable (EA), the model considered mixture, group, and the mixture × group interaction. Secondary variables were plasma tryptophan and oxytocin levels, PA, NA, VAS(+), and VAS(−). For plasma tryptophan and oxytocin levels, the models considered mixture, group, sample (morning, afternoon), and their interactions. For PA, NA, VAS(+), and VAS(−), the models considered mixture, group, time (0, t5, t6, t7), and their interactions.

We had no a priori hypotheses about gender or mixture × gender effects on EA. Yet, as women may be more susceptible to the mood effects of ATD, we added gender as a covariate to the analyses described in the previous paragraphs. We added order (T− first, B first) as a second covariate.

Significance was set at 0.05. Significant interaction terms were analyzed post hoc using simple contrasts, including Tukey-Kramer corrections for multiple comparisons. Results of HLM are reported using estimated least-squares means and standard errors of the mean (SEM). Cohen’s *d* was used to indicate effect sizes when comparing two means.

## Results

### Baseline mood

Morning QIDS scores did not vary significantly by mixture (*F*(1,37) = 0.11, *p* = 0.74, *d* = 0.11) and group (*F*_1,37_ = 3.07, *p* = 0.09, *d* = 0.58). The mixture × group interaction was significant (*F*_1,37_ = 4.55, *p* = 0.04), but post hoc comparisons revealed no significant effects (all *ps >* 0.06). Notably, no participant scored >7 on the QIDS. For baseline PA, NA, VAS(+), and VAS(−), there were no significant effects of mixture, group, and the mixture × group interaction (see Table 2).

**Table 2:** *F* values for the effects of mixture, group, and mixture × group on baseline mood

	PA	NA	VAS(+)	VAS(−)
Mixture	0.15	0.31	0.03	<0.01
Group	0.04	3.33	0.84	0.17
Mixture × group	1.36	0.07	0.50	0.57

*PA* positive affect, *NA* negative affect, *VAS(+)* visual analogue scale positive mood, *VAS(−)* visual analogue scale negative mood

### Empathic accuracy

One participant thought he recognized one target, and one thought he recognized two targets. We discarded the data pertaining to these participant/target combinations. The two sets of film clips generated similar mean levels of EA (set 1 v. set 2: 0.61 [SEM 0.02] v. 0.62 [SEM 0.02], *t*(1547) = −0.31, *p* > 0.75).

The main model revealed no significant effects for group (*F*(1,40) = 0.08, *p* = 0.78, *d* = 0.09), mixture (*F*(1,1548) = 0.60, *p* = 0.44, *d* = 0.04), and mixture × group (*F*(1,1548) = 0.01, *p* = 0.90). This suggested that ATD did not significantly alter EA in either group.

As EA was higher for positive clips (mean *r* = 0.72) than for negative clips (mean *r* = 0.51) (*F*(1,1548) = 149.9, *p* < 0.001, *d* = 0.62), we examined whether clip valence moderated the effect of ATD on EA. The mixture × valence interaction (*F*(1,1548) = 0.08, *p* = 0.78) and the mixture × group × valence interaction (*F*(1,1548) = 2.88, *p* = 0.09) were not significant.

As EA was higher for female targets (mean *r* = 0.64) than for male targets (mean *r* = 0.51) (*F*(1,1551) = 29.9, *p* < 0.001, *d* = 0.28), we examined whether target gender moderated the effect of ATD on EA. The mixture × target gender interaction (*F*(1,1548) = 0.02, *p* = 0.90) and the mixture × group × target gender interaction (*F*(1,1548) = 1.26, *p* = 0.26) were not significant. We also considered target expressivity as a moderator of the effect of ATD on EA. Results (not shown) were similar to the results where target gender was included as moderator.

All analyses were repeated for the two FH groups separately, for the two participants genders separately, for the two target genders separately, and for the positive and negative clips separately. The effects of mixture or group × mixture were never significant (all *p*s > 0.13). This suggests the study was not underpowered. In short, we did not find any effects of ATD on EA.

### Tryptophan and oxytocin

Two morning blood samples were not obtained. For tryptophan, the mixture × sample interaction was significant (*F*(1,118) = 443.41, *p* < 0.001). Total tryptophan levels decreased from 61.6 μM (SEM 2.1) to 7.1 μM (SEM 2.1) following T−, *t*(116) = 20.16, *p* < 0.0014, *d* = 3.74, and increased from 61.0 μM (SEM 2.1) to 84.1 μM (SEM 2.1) following B, *t*(116) = −8.67, *p* < 0.001, *d* = 1.61. The minimum decrease following T− was 77 % (mean 89 %). As the mixture × group × sample interaction was not significant (*F*(1,118) = 1.78, *p* = 0.18), the effect of ATD on total tryptophan levels was similar in the two FH groups.

Nearly two thirds of the oxytocin values were below the detection threshold of 1.5 pM. The number of missing values did not differ significantly between the morning and afternoon (*Χ*^2^ (1, *N* = 101) = 4.62, *p* = 0.20). To examine the effects of ATD on oxytocin, the 13 participants with at least one detectable value per test day (5 FH+ men; 3 FH+ women; 2 FH− men; 3 FH− women) were included in the analyses with their undetectable values conservatively recoded to 1.5 pM. The mixture × sample interaction was significant (*F*(1,39) = 6.00, *p* = 0.02). Oxytocin levels significantly decreased from morning to afternoon following T− (*t*(33) = 5.36, *p* < 0.001, *d* = 1.87) but not following B (*t*(33) = 2.17, *p* = 0.15, *d* = 0.76). The mixture × group × sample interaction was not significant (*F*(1,39) = 1.66, *p* = 0.21).

In sum, ATD was effective in reducing total tryptophan levels and also appeared to reduce oxytocin levels. The two FH groups were not differentially affected.

### Oxytocin and EA

As ATD reduced oxytocin levels in the participants with usable data, we explored whether their afternoon oxytocin levels were predictive of EA. This was not found (*F*(1,38) = 0.70, *p* = 0.41, *d* = 0.27). The correlation *r* between afternoon oxytocin levels and mean EA across film clips was 0.01, *p* = 0.99.

### Mood state

Table 3 summarizes the effects of mixture, group, time, and their interactions on mood.

**Table 3:** *F* values for the effects of mixture, group, time, and their interactions on mood

	PA	NA	VAS(+)	VAS(−)
Gender	1.84	<0.01	0.70	0.38
Order	3.31	0.22	1.74	0.07
Mixture	8.56**	0.18	1.65	0.25
Group	0.25	1.82	0.01	0.76
Time	11.84***	3.74*	7.64***	5.31**
Mixture × group	7.04**	0.16	4.23*	0.02
Mixture × time	0.25	0.85	0.57	0.73
Group × time	0.57	0.83	1.98	2.70*
Mixture × group × time	0.17	0.51	0.36	0.15

*PA* positive affect, *NA* negative affect, *VAS(+)* visual analogue scale positive mood, *VAS(−)* visual analogue scale negative mood

*p < 0.05; **p < 0.01; ***p < 0.001

#### PA

There was a significant main effect for mixture. The mixture × group interaction was also significant. In FH+ participants, PA was lower on the T− day (*M* = 2.45, SEM = 0.14) than on the B day (*M* = 2.66, SEM = 0.14 (*t*(266) = 3.85, *p* < 0.001, *d* = 0.47). In FH− participants, PA did not differ between the T− day (*M* = 2.46, SEM = 0.14) and the B day (*M* = 2.47, SEM = 0.14) (*t*(266) = 0.19, *p* > 0.99, *d* = 0.02). There was also a significant main effect for time. PA was higher at t0 (*M* = 2.66, SEM = 0.10) than at t6 (*M* = 2.51, SEM = 0.10) (*t*(266) = 2.81, *p* = 0.03, *d* = 0.34) and t7 (*M* = 2.34, SEM = 0.10) (*t*(266) = 5.79, *p* < 0.001, *d* = 0.71). More importantly, however, there were no significant effects of mixture × time and mixture × group × time, indicating that levels of PA did not change differently on the T− day compared to the B day.

#### NA

There was only a significant main effect for time. NA was significantly higher at t0 (*M* = 1.11, SEM = 0.02) than at t6 (*M* = 1.05, SEM = 0.02) (*t*(266) = 3.22, *p* = 0.008, *d* = 0.39).

#### VAS(+)

The mixture × group interaction was significant. In FH+ participants, VAS (+) was lower on the T− day (*M* = 42.99, SEM = 2.92) than on the B day (*M* = 46.41, SEM = 2.92) (*t*(266) = 2.75, *p* = 0.03, *d* = 0.34). There was also a significant main effect for time. VAS(+) was significantly higher at t0 (*M* = 49.33, SEM = 2.16) than at t5 (*M* = 45.36, SEM = 2.16) (*t*(266) = 3.18, *p* = 0.008, *d* = 0.39), t6 (*M* = 44.39, SEM = 2.16) (*t*(266) = 3.97, *p* < 0.001, *d* = 0.49), and t7 (*M* = 44.31, SEM = 2.16) (*t*(266) = 4.03, *p* < 0.001, *d* = 0.49). However, there were no significant effects of mixture × time and mixture × group × time, indicating that levels of positive mood did not change differently on the T− day compared to the B day.

#### VAS(*−*)

There was a significant main effect for time. VAS(−) was significantly lower at t0 (*M* = 5.76, SEM = 0.90) than at t7 (*M* = 7.02, SEM = 0.90) (*t*(266) = −2.66, *p* = 0.04, *d* = 0.33). The group × time interaction was also significant. However, post hoc contrasts between morning and afternoon VAS(−) levels were not significant for either FH group (all *p*s *>* 0.06).

In sum, mood state varied somewhat over the course of the test days. However, the time pattern did not differ by mixture. In other words, ATD did not influence mood.

## Discussion

We found no significant effect of ATD on empathic accuracy (EA) in individuals with (FH+) and without (FH−) a family history of MDD, though ATD significantly reduced blood oxytocin levels.

Previous research has indicated that, in healthy never-depressed individuals, ATD can have a negative impact on aspects of social functioning other than EA, such as facial emotion recognition (FER) (Harmer et al. 2003) and cooperation during the prisoner’s dilemma game (Wood et al. 2006). These findings suggest that ATD can impair performance on tasks that require participants to respond to limited social information. In contrast, we found that ATD did not impair performance on a task that involves responding to social information presented in a more dynamic, multimodal, and realistic way. While ATD may have impaired the processing of negative facial expressions (cf. Harmer et al. 2003; aan het Rot et al. 2010; van der Veen et al. 2007), participants in our study may have been able to maintain good EA because other (e.g., visual plus auditory) information was available. Moreover, there is evidence from experimental studies that ecstasy, an acute serotonin releaser, may have few effects on cognitive empathy even though more affective forms of empathy can improve (Hysek and Liechti 2012; Hysek et al. 2014; Schmid et al. 2014). In other words, in healthy, never-depressed individuals acute reductions in brain serotonin may negatively impact affective empathy but not cognitive empathy (Hysek et al. 2014; Schmid et al. 2014).

Both FH groups maintained good EA during ATD. This was unexpected as previous studies indicated that FH+ individuals may be more susceptible to the effects of ATD on the processing of socio-affective information (Feder et al. 2011; Firk and Markus 2008; van der Veen et al. 2007). This susceptibility has been suggested to indicate a risk factor for the development of depression. Mean levels of EA were comparable in the two FH groups and similar to levels seen in previous studies (aan het Rot and Hogenelst 2014; Zaki, Bolger, and Ochsner 2008). Therefore, alterations in EA induced by low serotonin might not play a role in the elevated MDD risk of FH+ individuals compared to FH− individuals.

The absence of an effect of ATD on EA cannot be attributed to an insufficient depletion of tryptophan. Five hours after ingestion of the T− mixture, total plasma levels of tryptophan were on average reduced by 89 %. This is similar to previous studies (e.g., Feder et al. 2011; Firk and Markus 2008; van der Veen et al. 2007). A lack of statistical power was also deemed an unlikely explanation for the lack of a significant effect of ATD on EA. We repeated the planned analyses of the effect of ATD on EA in the two FH groups separately (*n* = 20 per group), and there was no significant effect of ATD on EA in either group. Similarly, we repeated the analyses in the two genders separately (*n* = 20 per gender) and there was no significant effect of ATD on EA in either gender. The effect size *d* was never greater than 0.11. Moreover, our sample size was comparable with that of previous FH+ studies on the effects of ATD on socio-affective processing (Feder et al. 2011; Firk and Markus 2008; van der Veen et al. 2007).

In a subgroup of 13 participants with usable data, ATD significantly reduced circulating oxytocin levels. As oxytocin levels were often below the detection threshold, the finding that ATD reduced oxytocin requires replication. Nevertheless, the finding appears to be in line with previous studies in which stimulation of the brain serotonin system increased oxytocin release (e.g., Jorgensen et al. 2003; Lee, Garcia, van de Kar, Hauger, and Coccaro 2003; Ramos et al. 2013; Thompson et al. 2007). Our results extend these findings by suggesting that an acute reduction in brain serotonin may decrease oxytocin release.

The finding that ATD reduced circulating oxytocin levels but not EA appears to be at odds with a previous study in which intranasal oxytocin administration improved EA in individuals with higher levels of autism spectrum traits (Bartz et al. 2010). This study suggests there is a positive association between brain levels of oxytocin and EA. However, this effect was not seen in individuals with lower levels of autism spectrum traits, and we do not know the levels of these traits in our participants. Moreover, while brain levels of oxytocin are likely altered when oxytocin is administered intranasally and blood levels of oxytocin may be altered by ATD, it is unclear whether brain levels of oxytocin were altered by ATD. Basal concentrations of oxytocin in blood plasma and cerebrospinal fluid may not be correlated (Striepens et al. 2013).

We had expected FH+ individuals in particular to be sensitive to ATD-induced mood lowering (Booij et al. 2003; van der Veen et al. 2007), but ATD did not significantly lower mood in either FH group. Nevertheless, other ATD studies have also found no difference in mood change between FH+ and FH− individuals (e.g., Ellenbogen, Young, Dean, Palmour, and Benkelfat 1999; Feder et al. 2011). It has previously been suggested that FH+ participants with no personal psychiatric history and who do not drop out of the study constitute a group with low susceptibility to ATD-induced mood change (Ellenbogen et al. 1999). In the present study, there were few exclusions and dropouts, but it remains possible that FH+ individuals with a psychiatric history were not screened because they were not recruited.

### Limitations

The family history method we used provides insight in inter-individual variability in familial MDD load. However, it does not yield diagnoses in family members (Andreasen et al. 1977). FH+ individuals with multiple family members diagnosed with MDD may respond differently to ATD than FH+ individuals with a single affected family member. We could not formally test this.

Differential effects of ATD in our study versus previous studies assessing the effects of ATD on responses to socio-affective information and mood in FH+ individuals (Feder et al. 2011; Firk and Markus 2008; van der Veen et al. 2007) may be explained by between-study differences in MDD patient characteristics such as symptom levels, number of experienced depressive episodes, and medication status. We did not collect information on these characteristics nor did several previous studies (e.g., van der Veen et al. 2007; Firk and Markus 2008). This precludes comparing our study with these previous studies. An exception is a study by Feder et al. (2011), who only included patients with recurrent or chronic MDD who had been prescribed antidepressant medication. Future studies may include more elaborate patient information.

By asking participants to rate how targets in the video clips felt, the EA task is thought to assess cognitive empathy. While the task could have been adapted to assess the more affective aspects of empathy, by asking participants to rate how they felt while listening to the targets, this was not done. Moreover, though the EA task uses stimuli with high ecological validity and performance is impaired in clinical groups with social deficits (Lee et al. 2011; Ripoll et al. 2013), the predictive value of the task for everyday social functioning has not been determined. This limits the extent to which the results of our study are relevant for real life. However, FER task performance may also be unrelated to everyday social functioning (Janssens et al. 2012).

ATD did not significantly lower mood or alter EA. Yet, EA may have been affected by ATD if there had been effects on mood. It therefore remains an open question whether a sample of FH+ individuals who do experience mood changes after ATD would also show effects on EA.

### Suggestions for future studies

Remitted MDD patients are more sensitive to the mood-lowering effect of ATD than never-depressed individuals (Young 2013). Moreover, in remitted MDD patients, ATD has previously been shown to impair the recognition of happy facial expressions (Hayward et al. 2005). Given their susceptibility to mood lowering and impaired FER after ATD, compared to FH+ individuals, remitted MDD patients may be more susceptible to ATD-induced impairments in EA. This could be assessed in a future study and may inform about the neurobiology underlying impaired empathy in MDD (Schreiter et al. 2013).

Depressed individuals’ interpersonal difficulties are especially likely to emerge in the context of intimate relationships (Segrin and Flora 2000). In a future study involving FH+ individuals, ATD could be combined with a laboratory paradigm that involves assessment of EA during an interaction in which couples are asked to collaborate. This has previously been done in a study that found depressed individuals to have worse EA than nondepressed individuals (Gadassi et al. 2011).

### Conclusion

Previous research indicates that acute reductions in brain serotonin can impair behavioral responses to static facial expressions in healthy individuals, including those at familial risk for MDD (aan het Rot et al. 2010; Harmer et al. 2003; van der Veen et al. 2007). Our results suggest that when social stimuli are more realistic, even though brain oxytocin levels may be reduced, behavioral responses may remain intact.

The present study does not provide evidence for a role of serotonin in regulating empathic abilities in never-depressed individuals. Additional experiments are needed to determine whether serotonin regulates empathic abilities in individuals with MDD and whether this is mediated by oxytocin.

## Electronic supplementary material

ESM 1 Details of the screening phase and psychometric properties of the PANAS and VAS (DOCX 23 kb)

## References

[CR1] aan het Rot M, Hogenelst K (2014). The influence of affective empathy and autism spectrum traits on empathic accuracy. PLoS One.

[CR2] aan het Rot M, Coupland N, Boivin DB, Benkelfat C, Young SN (2010). Recognizing emotions in faces: effects of acute tryptophan depletion and bright light. Am. J. Psychol (Oxford, England).

[CR3] Ambadar Z, Schooler JW, Cohn JF (2005). Deciphering the enigmatic face: the importance of facial dynamics in interpreting subtle facial expressions. Psychol Sci.

[CR4] Andreasen NC, Endicott J, Spitzer RL, Winokur G (1977). The family history method using diagnostic criteria. reliability and validity. Arch Gen Psychiatry.

[CR5] Bartz JA, Zaki J, Bolger N, Hollander E, Ludwig NN, Kolevzon A, Ochsner KN (2010). Oxytocin selectively improves empathic accuracy. Psychol Sci.

[CR6] Bedi G, Hyman D, de Wit H (2010). Is ecstasy an “empathogen”? effects of +/−3,4-methylenedioxymethamphetamine on prosocial feelings and identification of emotional states in others. Biol Psychiatry.

[CR7] Bond AJ, James DC, Lader MH (1974). Physiological and psychological measures in anxious patients. Psychol Med.

[CR8] Booij L, Van der Does AJ, Riedel WJ (2003). Monoamine depletion in psychiatric and healthy populations: review. Mol Psychiatry.

[CR9] Bourke C, Douglas K, Porter R (2010). Processing of facial emotion expression in major depression: a review. Aust. N. Z. J. Psychiatry.

[CR10] Crockett MJ, Clark L, Tabibnia G, Lieberman MD, Robbins TW (2008). Serotonin modulates behavioral reactions to unfairness. Science (New York, NY).

[CR11] de Jong WH, Smit R, Bakker SJ, de Vries EG, Kema IP (2009). Plasma tryptophan, kynurenine and 3-hydroxykynurenine measurement using automated on-line solid-phase extraction HPLC-tandem mass spectrometry. J. Chromatogr. B Anal. Technol. Biomed. Life Sci.

[CR12] Demenescu LR, Kortekaas R, den Boer JA, Aleman A (2010). Impaired attribution of emotion to facial expressions in anxiety and major depression. PLoS One.

[CR13] Dumont GJ, Sweep FC, van der Steen R, Hermsen R, Donders AR, Touw DJ, . . . Verkes RJ (2009) Increased oxytocin concentrations and prosocial feelings in humans after ecstasy (3,4-methylenedioxymethamphetamine) administration. Social Neuroscience, 4(4), 359–366. 10.1080/17470910802649470 10.1080/17470910802649470 19562632

[CR14] Ellenbogen MA, Young SN, Dean P, Palmour RM, Benkelfat C (1996). Mood response to acute tryptophan depletion in healthy volunteers: sex differences and temporal stability. Neuropsychopharmacol Off Pub Am College of Neuropsychopharmacol.

[CR15] Ellenbogen MA, Young SN, Dean P, Palmour RM, Benkelfat C (1999). Acute tryptophan depletion in healthy young women with a family history of major affective disorder. Psychol Med.

[CR16] Feder A, Skipper J, Blair JR, Buchholz K, Mathew SJ, Schwarz M, . . . Charney DS (2011). Tryptophan depletion and emotional processing in healthy volunteers at high risk for depression. Biological Psychiatry, 69(8), 804–807. 10.1016/j.biopsych.2010.12.033 10.1016/j.biopsych.2010.12.033 PMC3941748 21377656

[CR17] Firk C, Markus CR (2008). Effects of acute tryptophan depletion on affective processing in first-degree relatives of depressive patients and controls after exposure to uncontrollable stress. Psychopharmacology.

[CR18] First MB, Spitzer RL, Gibbon M, Williams JBW (2002). Structured clinical interview for DSM-IV-TR axis I disorders, research version, patient edition. (SCID-I/P).

[CR19] Gadassi R, Mor N, Rafaeli E (2011). Depression and empathic accuracy in couples: an interpersonal model of gender differences in depression. Psychol Sci.

[CR20] Gross JJ, John OP (1997). Revealing feelings: facets of emotional expressivity in self-reports, peer ratings, and behavior. J Pers Soc Psychol.

[CR21] Hames JL, Hagan CR, Joiner TE (2013). Interpersonal processes in depression. Annu Rev Clin Psychol.

[CR22] Harmer CJ, Rogers RD, Tunbridge E, Cowen PJ, Goodwin GM (2003). Tryptophan depletion decreases the recognition of fear in female volunteers. Psychopharmacology.

[CR23] Hayward G, Goodwin GM, Cowen PJ, Harmer CJ (2005). Low-dose tryptophan depletion in recovered depressed patients induces changes in cognitive processing without depressive symptoms. Biol Psychiatry.

[CR24] Hogenelst K, Schoevers RA, & aan het Rot M (2015) Studying the neurobiology of human social interaction: making the case for ecological validity. *Social Neuroscience*, 1–11. 10.1080/17470919.2014.994786 10.1080/17470919.2014.994786 25566795

[CR25] Hysek CM, & Liechti ME (2012) Effects of MDMA alone and after pretreatment with reboxetine, duloxetine, clonidine, carvedilol, and doxazosin on pupillary light reflex. Psychopharmacology, 224(3), 363–376. 10.1007/s00213-012-2761-6 10.1007/s00213-012-2761-6 22700038

[CR26] Hysek CM. Schmid Y, Simmler LD, Domes G, Heinrichs M, Eisenegger C, . . . Liechti ME (2014) MDMA enhances emotional empathy and prosocial behavior. Social Cognitive and Affective Neuroscience, 9(11), 1645–1652. 10.1093/scan/nst161 10.1093/scan/nst161 PMC4221206 24097374

[CR27] Ickes W (1993). Empathic accuracy. J Pers.

[CR28] Janssens M, Lataster T, Simons CJ, Oorschot M, Lardinois M, van Os J, . . . GROUP (2012) Emotion recognition in psychosis: no evidence for an association with real world social functioning. Schizophrenia Research, 142(1–3), 116–121.10.1016/j.schres.2012.10.003 10.1016/j.schres.2012.10.003 23122740

[CR29] Jorgensen H, Riis M, Knigge U, Kjaer A, Warberg J (2003). Serotonin receptors involved in vasopressin and oxytocin secretion. J Neuroendocrinol.

[CR30] Klaassen T, Riedel WJ, van Someren A, Deutz NE, Honig A, van Praag HM (1999). Mood effects of 24-hour tryptophan depletion in healthy first-degree relatives of patients with affective disorders. Biol Psychiatry.

[CR31] Lee R, Garcia F, van de Kar LD, Hauger RD, Coccaro EF (2003). Plasma oxytocin in response to pharmaco-challenge to D-fenfluramine and placebo in healthy men. Psychiatry Res.

[CR32] Lee J, Zaki J, Harvey PO, Ochsner K, Green MF (2011). Schizophrenia patients are impaired in empathic accuracy. Psychol Med.

[CR33] Mann JJ (2013). The serotonergic system in mood disorders and suicidal behaviour. Philos. Trans. R. Soc. Lond. Ser. B Biol. Sci.

[CR34] Mannie ZN, Bristow GC, Harmer CJ, Cowen PJ (2007). Impaired emotional categorisation in young people at increased familial risk of depression. Neuropsychologia.

[CR35] Marsh AA, Finger EC, Buzas B, Soliman N, Richell RA, Vythilingham M, . . . Blair RJ (2006) Impaired recognition of fear facial expressions in 5-HTTLPR S-polymorphism carriers following tryptophan depletion. Psychopharmacology, 189(3), 387–394. 10.1007/s00213-006-0581-2 10.1007/s00213-006-0581-2 17013635

[CR36] Neumeister A, Konstantinidis A, Stastny J, Schwarz MJ, Vitouch O, Willeit M, . . . Kasper S (2002) Association between serotonin transporter gene promoter polymorphism (5HTTLPR) and behavioral responses to tryptophan depletion in healthy women with and without family history of depression. *Archives of General Psychiatry, 59*(7), 613–620. 10.1001/archpsyc.59.7.613 10.1001/archpsyc.59.7.613 12090814

[CR37] Nishizawa S, Benkelfat C, Young SN, Leyton M, Mzengeza S, de Montigny C, . . . Diksic M (1997) Differences between males and females in rates of serotonin synthesis in human brain. *Proceedings of the National Academy of Sciences of the United States of America, 94*(10), 5308–5313. 10.1073/pnas.94.10.5308 10.1073/pnas.94.10.5308 PMC24674 9144233

[CR38] Peeters FPML, Ponds RWHM, Vermeeren MTG (1996). Affectiviteit en zelfbeoordeling van depressie en angst. Tijdschift Voor Psychiatrie.

[CR39] Penton-Voak I, Allen T, Morrison ER, Gralewski L, Campbell N (2007). Performance on a face perception task is associated with empathy quotient scores, but not systemizing scores or participant sex. Personal Individ Differ.

[CR40] Ramos L, Hicks C, Kevin R, Caminer A, Narlawar R, Kassiou M, McGregor IS (2013). Acute prosocial effects of oxytocin and vasopressin when given alone or in combination with 3,4-methylenedioxymethamphetamine in rats: involvement of the V1A receptor. Neuropsychopharmacol Off Pub Am College of Neuropsychopharmacol.

[CR41] Ripoll LH, Zaki J, Perez-Rodriguez MM, Snyder R, Strike KS, Boussi A, . . . New A S. (2013) Empathic accuracy and cognition in schizotypal personality disorder. Psychiatry Research, 210(1), 232–241. 10.1016/j.psychres.2013.05.025 10.1016/j.psychres.2013.05.025 23810511

[CR42] Rush AJ, Trivedi MH, Ibrahim HM, Carmody TJ, Arnow B, Klein DN, . . . Keller MB (2003) The 16-item quick inventory of depressive symptomatology (QIDS), clinician rating (QIDS-C), and self-report (QIDS-SR): a psychometric evaluation in patients with chronic major depression. *Biological Psychiatry, 54*(5), 573–583. 10.1016/S0006-3223(02)01866-8 10.1016/s0006-3223(02)01866-8 12946886

[CR43] Schmid Y, Hysek CM, Simmler LD, Crockett MJ, Quednow BB, Liechti ME (2014). Differential effects of MDMA and methylphenidate on social cognition. J. Psychopharmacol (Oxford, England).

[CR44] Schreiter S, Pijnenborg GH, aan het Rot M (2013). Empathy in adults with clinical or subclinical depressive symptoms. J Affect Disord.

[CR45] Segrin C, Flora J (2000). Poor social skills are a vulnerability factor in the development of psychosocial problems. Hum Commun Res.

[CR46] Shamay-Tsoory SG, Aharon-Peretz J, Perry D (2009). Two systems for empathy: a double dissociation between emotional and cognitive empathy in inferior frontal gyrus versus ventromedial prefrontal lesions. Brain J. Neurol.

[CR47] Striepens N, Kendrick KM, Hanking V, Landgraf R, Wullner U, Maier W, Hurlemann R (2013). Elevated cerebrospinal fluid and blood concentrations of oxytocin following its intranasal administration in humans. Sci Rep.

[CR48] Sullivan PF, Neale MC, Kendler KS (2000). Genetic epidemiology of major depression: review and meta-analysis. Am J Psychiatry.

[CR49] Thompson MR, Callaghan PD, Hunt GE, Cornish JL, McGregor IS (2007). A role for oxytocin and 5-HT (1A) receptors in the prosocial effects of 3, 4 methylenedioxymethamphetamine (“ecstasy”). Neuroscience.

[CR50] van der Veen FM, Evers EA, Deutz NE, Schmitt JA (2007). Effects of acute tryptophan depletion on mood and facial emotion perception related brain activation and performance in healthy women with and without a family history of depression. Neuropsychopharmacol Off Pub Am College of Neuropsychopharmacol.

[CR51] Verhofstadt LL, Buysse A, Ickes W, Davis M, Devoldre I (2008). Support provision in marriage: the role of emotional similarity and empathic accuracy. Emotion (Washington, DC).

[CR52] Williams JHG, Perrett DI, Waiter GD, Pechey S (2007). Differential effects of tryptophan depletion on emotion processing according to face direction. Soc Cogn Affect Neurosci.

[CR53] Wood RM, Rilling JK, Sanfey AG, Bhagwagar Z, Rogers RD (2006). Effects of tryptophan depletion on the performance of an iterated prisoner’s dilemma game in healthy adults. Neuropsychopharmacol Off Pub Am College of Neuropsychopharmacol.

[CR54] Young SN (2013). The effect of raising and lowering tryptophan levels on human mood and social behaviour. Philos. Trans. R. Soc. Lond. Ser. B Biol. Sci.

[CR55] Young SN, Smith SE, Pihl RO, Ervin FR (1985). Tryptophan depletion causes a rapid lowering of mood in normal males. Psychopharmacology.

[CR56] Zaki J, Ochsner KN (2009). The need for a cognitive neuroscience of naturalistic social cognition. Ann N Y Acad Sci.

[CR57] Zaki J, Ochsner KN (2012). The neuroscience of empathy: progress, pitfalls and promise. Nat Neurosci.

[CR58] Zaki J, Bolger N, Ochsner KN (2008). It takes two: the interpersonal nature of empathic accuracy. Psychol Sci.

[CR59] Zaki J, Bolger N, Ochsner KN (2009). Unpacking the informational bases of empathic accuracy. Emotion (Washington, DC).

[CR60] Zaki J, Weber J, Bolger N, Ochsner KN (2009). The neural bases of empathic accuracy. Proc Natl Acad Sci U S A.

