# Supplementary material for: Empathic accuracy and oxytocin after tryptophan depletion in adults at risk for depression
Source: Psychopharmacology (Berl). 2015 Oct 13;233:111–20. doi: 10.1007/s00213-015-4093-9 (PMC4700075; doi:10.1007/s00213-015-4093-9)
Supplement: Supplementary file 1 — Details of the screening phase and psychometric properties of the PANAS and VAS (DOCX 23 kb) [file 213_2015_4093_MOESM1_ESM.docx]

**Supplementary material**

**Screening phase**

FH+ individuals were recruited via their affected family members. These probands with a diagnosis of MDD were informed about the study via their health-care provider (e.g., psychiatrist, clinical psychologist) or via advertisements. Probands were asked if they had never-depressed first-degree relatives motivated to participate in a study on the role of serotonin on mood and social behavior. A total of 148 probands responded. Following a brief explanation of the study, 45 probands were interested in receiving further information. We obtained written informed consent from 35 probands to contact their health-care provider and verify the diagnosis of MDD and absence of a lifetime history of (hypo) mania. We excluded 4 individuals who did not meet the diagnostic criteria and 3 individuals whose health care provider did not verify the diagnosis. One individual was lost to follow-up.

A total of 34 FH+ individuals were interested in receiving further information about the study. We obtained written informed consent from 26 individuals. We excluded 1 individual because of past panic disorder and 2 individuals were lost to follow-up. A total of 23 FH+ participants started the study.

FH- individuals were recruited using local advertisements and a paid participant pool of the University of Groningen. Thirty-seven FH- individuals were interested in receiving further information about the study. We obtained written informed consent from 24 individuals. We excluded 2 individuals because of a family history of suicide and 1 individual was lost to follow-up. A total of 21 FH+ participants started the study.

**Mood questionnaires**

Mood state was measured using a Dutch version of the Positive Affect and Negative Affect Schedule (PANAS) (56) and a list of Visual Analogue Scales (VAS) (57). The PANAS included 10 positive affect (PA) items and 10 negative affect (NA) items, all rated on a 5-point Likert-scale. For the PA items, a factor analysis revealed a one-factor structure and the Cronbach coefficient α was 0.91. For the NA items, there was insufficient variability to determine a factor structure or internal consistency. The average of the PA and NA scores were used to calculate PA and NA, respectively.

The VAS included 7 positive (+) mood items and 6 negative (-) mood items, all rated on unipolar scales represented by 10 cm long lines ranging from *not at all* to *extreme*. For the VAS(+) items, a factor analysis revealed a one-factor structure and the Cronbach coefficient α was 0.91. For the VAS(-) items, a two-factor structure emerged, with only ‘bored’ loading on the second factor. The Cronbach coefficient α for VAS(-) was 0.58 including and 0.64 excluding ‘bored’. We excluded VAS(-) ‘bored’ from subsequent analyses. The average of the 7 positive and 5 remaining negative item scores were used to calculate VAS(+) and VAS(-), respectively.
